# Ormeloxifene-induced unfolded protein response contributes to autophagy-associated apoptosis via disruption of Akt/mTOR and activation of JNK

**DOI:** 10.1038/s41598-018-20541-8

**Published:** 2018-02-02

**Authors:** Arindam Bhattacharjee, Mohammad Hasanain, Manoj Kathuria, Akhilesh Singh, Dipak Datta, Jayanta Sarkar, Kalyan Mitra

**Affiliations:** 1 Electron Microscopy Unit, Sophisticated Analytical Instrument Facility, CSIR-Central Drug Research Institute, Sector-10, Jankipuram Extension, Lucknow, 226 031 India; 2 Biochemistry Division, CSIR-Central Drug Research Institute, Sector-10, Jankipuram Extension, Lucknow, 226 031 India; 3 Academy of Scientific and Innovative Research, Chennai, 600113 India

## Abstract

Autophagy, a regulated nutrient recycling program can affect both cell survival and cell death. Here, we show that Ormeloxifene (ORM), a selective estrogen receptor modulator approved for oral contraceptive use induces autophagic flux in ovarian cancer cells, which is activated by an ER stress response upstream of autophagy. The ER stress response is characterized by activation of IRE1α, PERK and ATF6 and is under regulation of JNK. Pharmacological inhibition of either autophagy or ER stress increased cell survival, as did silencing of autophagy proteins LC3 and Beclin 1, implying that ORM-induced autophagy is pro-death in nature. Ultrastructural observations of treated cells confirmed stages of autophagic maturation. Caspase-dependent apoptosis succeeded these events and was characterized by generation of reactive oxygen species and disruption of mitochondrial membrane potential. A concomitant inhibition of the Akt/mTOR axis was also observed with possible regulation of Akt by ORM. ORM inhibited tumor growth in ovarian xenograft model and displayed autophagic activity. In summary, *in vitro* and *in vivo* results reveal that ORM induces autophagy-associated cell death to attenuate proliferation of ovarian cancer cells. Our results demonstrate that using ORM in combination with ER stress and autophagy modulators could offer better therapeutic outcome in ovarian cancer.

## Introduction

Ovarian cancer is one of the leading gynecological malignancies among women in terms of mortality^1^. Diagnosis and treatment is complicated by late onset of symptoms and resistance to standard chemotherapeutics. Novel, safer drugs with good bioavailability for better therapeutic outcome can alleviate persisting problems of side effects and resistance. Selective estrogen receptor modulators (SERMs) are characterized by their diverse effect (anti-estrogenic or pro-estrogenic) against estrogen receptors in different tissues and are widely employed in cancer treatment. Ormeloxifene (ORM), an orally active non-steroidal SERM is clinically available in India as a contraceptive and is lately being investigated for its anticancer potential. A phase II clinical trial of ORM was conducted for advanced refractory breast cancer, which resulted in a 40.5% response rate^2^, followed by phase III trials. Previous *in vitro* and *in vivo* studies report that ORM induces apoptosis in breast and head and neck cancer, chronic myelegenous leukemia and ovarian cancer^3–6^. Cell cycle arrest, inactivation of Akt-mTOR and involvement of MAPKs have also been reported in ORM-mediated apoptosis^3–5^. However, here we report for the first time severe ER stress and autophagy associated cell death with ORM treatment. We have studied the role of autophagy in modulating cell death and present a more pharmacologically relevant mechanism of action of ORM, which might prove beneficial for a combination therapeutic approach. Macroautophagy (autophagy hereafter) is a catabolic process that recycles nutrients across the cell in double membrane vesicles (autophagosomes) that fuse with lysosomes for bulk degradation of cargo. Autophagy is considered to be a double-edged sword in cancer since depending on the cellular milieu, it can play either a pro-death or pro-survival role. While autophagic cell death, or ‘type-II cell death’, enhances the efficacy of anti-cancer drugs, a pro-survival role contributes to chemoresistance^7^. Previous studies have pointed out a pro-survival role of autophagy in imparting resistance against therapies that target the estrogen receptor (EsR) in estrogen receptor positive breast cancer^8^. Specifically, it was shown that this resistance was mediated by overexpression of GRP78/BiP (glucose-regulated protein-78 kDa), an ER chaperone, that in turn, activated autophagy and inhibited apoptosis. However, here we show that inhibiting autophagic progression by pharmacological and genetic methods in both EsR+ ve (OVCAR-3) and EsR –ve (PA-1) ovarian cancer cells effectively decreased apoptosis, indicating that ORM-induced autophagy has a pro-death function. We also show that ORM-induced autophagy is caused by endoplasmic reticulum (ER) stress and unfolded protein response (UPR), through the activation of transmembrane ER stress sensors PERK (PKR-like ER kinase), IRE1(inositol-requiring enzyme 1) and ATF6 (activating transcription factor 6). Autophagy induced by ORM show evidence of mitophagy as well as ER-phagy, and finally leads to apoptosis. Finally, ORM-induced autophagy is regulated by activation of the c-Jun N-terminal kinase (JNK), along with the disruption of the cellular Akt-mTOR axis. Autophagy modulation is emerging as a viable approach for anticancer therapy, and clinical trials for several such drugs are underway^9^. Additionally, available literature suggests that autophagy may play an important role in ovarian cancer^10^. The present study describes a mechanism of ORM action in ovarian cancer cells that can be exploited in combination with other drugs that work by modulating the autophagy or UPR pathway.

## Results

### ORM induces autophagic flux

**Figure 1:**
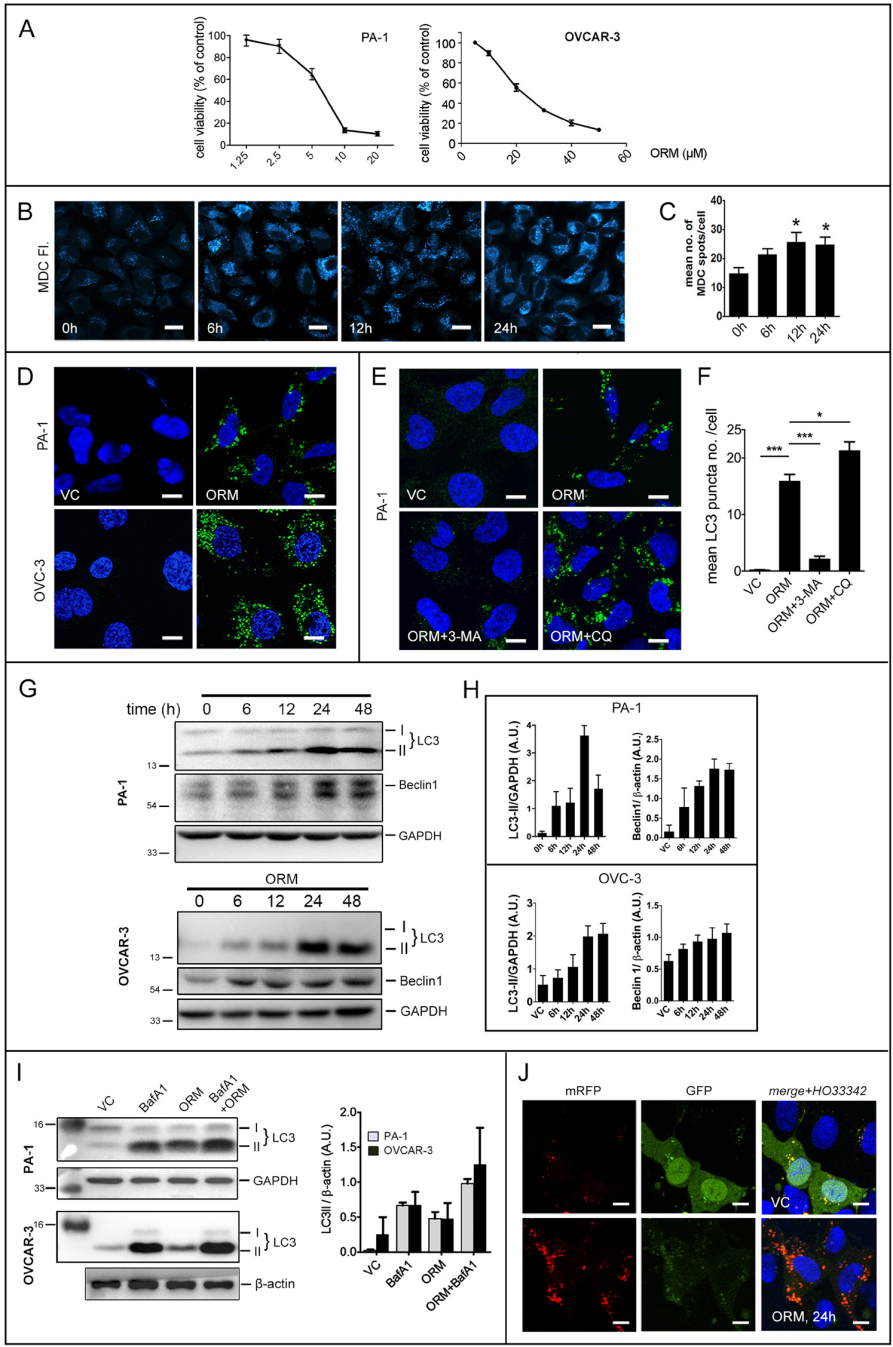
ORM induces autophagic flux in ovarian cancer cells PA-1 and OVCAR-3. (**A**) Dose-response curve of ORM in ovarian cancer cell lines PA-1 and OVCAR-3. (**B**) PA-1 cells were stained with MDC, a marker of acidic cellular compartments after treatment with indicated time periods with IC_50_ dose of ORM. (**C**) quantification of MDC-positive dots from (**B**) as described in Methods. (**D**) Confocal microscopy images of PA-1 and OVCAR-3 cells treated with IC_50_ dose of ORM and immunostained with LC3 (an autophagosome marker). (**E**) PA-1 cells were pre-treated with or without 3-MA (class III PI3K inhibitor; 2.5 mM) or CQ (lysosomal fusion inhibitor; 10 μM), immunostained with LC3 and imaged in confocal microscope. (**F**) Quantification of LC3 puncta from data such as (**E**) as described in Methods. (**G**) PA-1 and OVCAR-3 cells were treated with ORM (IC_50_ dose) for indicated time-points and probed for autophagy markers LC3 and Beclin 1, quantified in (**H**). (**I**) PA-1 and OVCAR-3 cells were pre-treated or not with lysosomal fusion inhibitor BafA1 (100 nM; 2 h) to inhibit fusion of autophagosomes with lysosomes. LC3-II conversion was observed with immunoblotting and quantified. (**J**) PA-1 cells were transfected with tfLC3 (as described in Methods), treated with ORM for 24 h and imaged in confocal microscope. Scale bars = 20 μm (**B**); 10 μm (**D**).

ORM-treatment decreased PA-1 and OVCAR-3 population in a dose-dependent manner (mean IC_50_ of ~7.5 μmol/L for PA-1 and ~18.8 μmol/L for OVCAR-3; doses used in subsequent experiments) (Fig. 1A). We investigated whether ORM has the potential to induce autophagy, and whether the autophagy has a role in its anti-proliferative action. A rapid way of assesing autophagic activity is to stain cells with the acidotropic marker monodansylcadaverine (MDC)^11^. Our initial data with PA-1 stained with MDC showed a significant increase in MDC stained vesicles with treatment time (Fig. 1B,C). Since MDC is not an absolute marker for autophagosomes^12^, we performed immunofluorescence and Western blotting to confirm autophagy. Immunostaining for LC3 (microtubule associated protein light chain 3; MAPLC3), a phagophore membrane component, gives a spatial information of autophagosomes inside the cell^13^. ORM significantly increased cytosolic LC3 puncta 24 h post-treatment (Fig. 1D). We found this number to decrease drastically when 3-MA (a type-III PI3k inhibitor and often used to inhibit Vps34 complex in autophagy initiation) was added, indicating ORM-induced autophagy follows the canonical macroautophagy pathway through the Beclin1-Vps34 initiation step (Fig. 1E,F). During autophagy, LC3-I (unconjugated form of LC3) is conjugated with phosphatidylethanolamine (PE) to form LC3-II by the action of Atg3/Atg7 and thereafter associates with autophagosomal membranes^14^. After fusion with lysosomes, LC3-II is degraded. Owing to this, LC3-II turnover is a reliable tool to measure autophagic flux^12^. Here, we found steadily increased LC3-II turnover in both PA-1 and OVCAR-3, the highest expression being at 24 h which reduced thereafter. Not surprisingly, Beclin 1 (a member of Beclin 1-Vps34-Vps15 core complex involved in initiation of autophagy^15^) was also found upregulated in a time-dependent manner (Fig. 1G,H); and Atg5 (Autophagy-related 5; initiates formation of and localizes to nascent phagophores) also followed the same trend (data not shown).

To verify whether LC3-II turnover was due to simply increased turnover of autophagosomes (true autophagic flux) or a blocking of autophagosome-lysosome fusion, we performed two separate experiments. First, the vacuolar ATPase inhibitor bafilomycin A1 (BafA1) was used since it prevents lysosomal fusion by raising lysosomal pH^16^. An increase in LC3-II turnover for ORM treatment in presence of BafA1 compared to BafA1 alone would indicate turnover of new autophagosomes, while unaltered LC3-II pattern between BafA1 and BafA1+ ORM would signify a block in the lysosomal fusion process by the drug. Increased LC3-II turnover was found in BafA1+ ORM treatment compared with BafA1 alone, implying that ORM induces true autophagic flux and does not alter the lysosomal fusion (Fig. 1I). Chloroquine (CQ), another fusion inhibitor, raised number of LC3 puncta compared to ORM, when observed microscopically (Fig. 1E,F).

Secondly, the tandem mRFP-GFP-LC3 (tfLC3)^13,17^ was used to visually determine fusion events, as GFP fluorescence of this fusion construct is quenched in the acidic environment of the autolysosome while being intact in nascent autophagosomal/cytosolic environment. A major advantage of this method is that it allows determination of autophagic flux without use of any additional inhibitors. Confocal imaging of ORM-treated PA-1 cells expressing tfLC3 revealed distinct red (mRFP) autolysosomal puncta (similar to amino acid starvation; data not shown) while VC cells had diffuse cytosolic distribution of GFP and mRFP fluorescence (Fig. 1J). This, along with previous observations, demonstrate that ORM induces true autophagic flux.

### TEM observations reveal an association of ER, mitochondria and autophagosomes after ORM treatment

**Figure 2:**
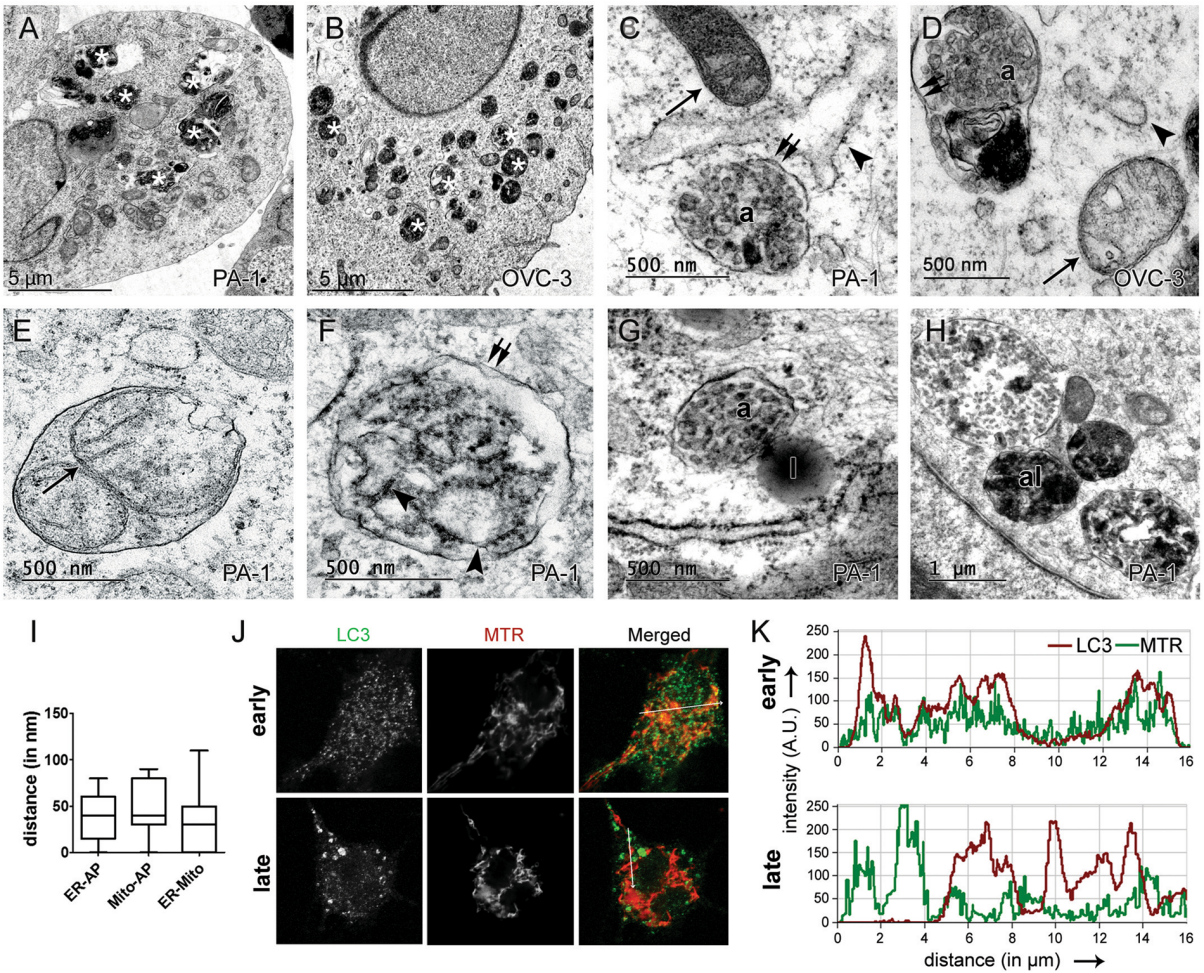
Involvement of ER and mitochondria in ORM-induced autophagy. Representative transmission electron micrographs of (**A**) PA-1 and (**B**) OVCAR-3 cells post-treatment with IC_50_ dose of ORM show numerous electron-dense autophagosomes (asterisks). (**C**) PA-1, and (**D**) OVCAR-3 cells early stages post-treatment (<3 h) showing a close association between ER (arrowheads), mitochondria (arrows) and autophagosomes [a]. (**E**) Engulfment of mitochondria, and (**F**) rough ER by autophagosomes observed in PA-1 cells. (**G**) Fusion of autophagosome with electron-dense lysosome [l] and, (**H**) formation of autolysosomes [al] in PA-1. (**I**) Calculation of mean distance between ER, mitochondria and autophagosomes in early stages of treatment (<3 h). (**J**) PA-1 cells treated with ORM for 3 h (early) and 24 h (late) were immunostained with LC3 and counterstained with Mitotracker Red (MTR). (**K**) Co-localization of MTR and LC3 signal observed in early stages (drag-in-image) while absent in late stages. Double arrows = double membraned structures.

Cellular ultrastructure of both PA-1 and OVCAR-3 revealed abundant autophagosomes and autophagosome-lysosome fusion events followed by autolysosome formation (Fig. 2A,B,G,H). Interestingly, early (1–3 h) observations of ORM-treated PA-1 cell ultrastructure revealed nascent autophagosomes closely juxtaposed with ER and mitochondria, with a median distance <100 nm in both instances (Fig. 2C,D,I). We also observed autophagosomes with engulfed mitochondria and mitochondrial remnants as well as rough ER, thus revealing incidences of mitophagy and ER-phagy (Fig. 2E,F) furthermore providing early indication of possible mitochondrial and ER stress resulting in engulfment of damaged ER and mitochondria by autophagosomes. The ER, mitochondria, golgi and plasma membrane as well as ER-mitochondria contact sites have been cited as autophagosome nucleation sites^18,19^. To differentiate the autophagosome-mitochondrial association with increasing treatment time, early (<3 h) and late (24 h) treatment of ORM was given to PA-1 cells, were immunostained with LC3 and counterstained with Mitotracker Red (MTR; a mitochondrial marker). A distinct co-localization between LC3 and MTR signal was observed in early stages post-treatment (<3 h), which declined afterwards (24 h) (Fig. 2J,K). The data indicate a possible interaction of mitochondria and autophagosomes at early stages of formation.

### ORM promotes apoptosis through mitochondrial pathway

**Figure 3:**
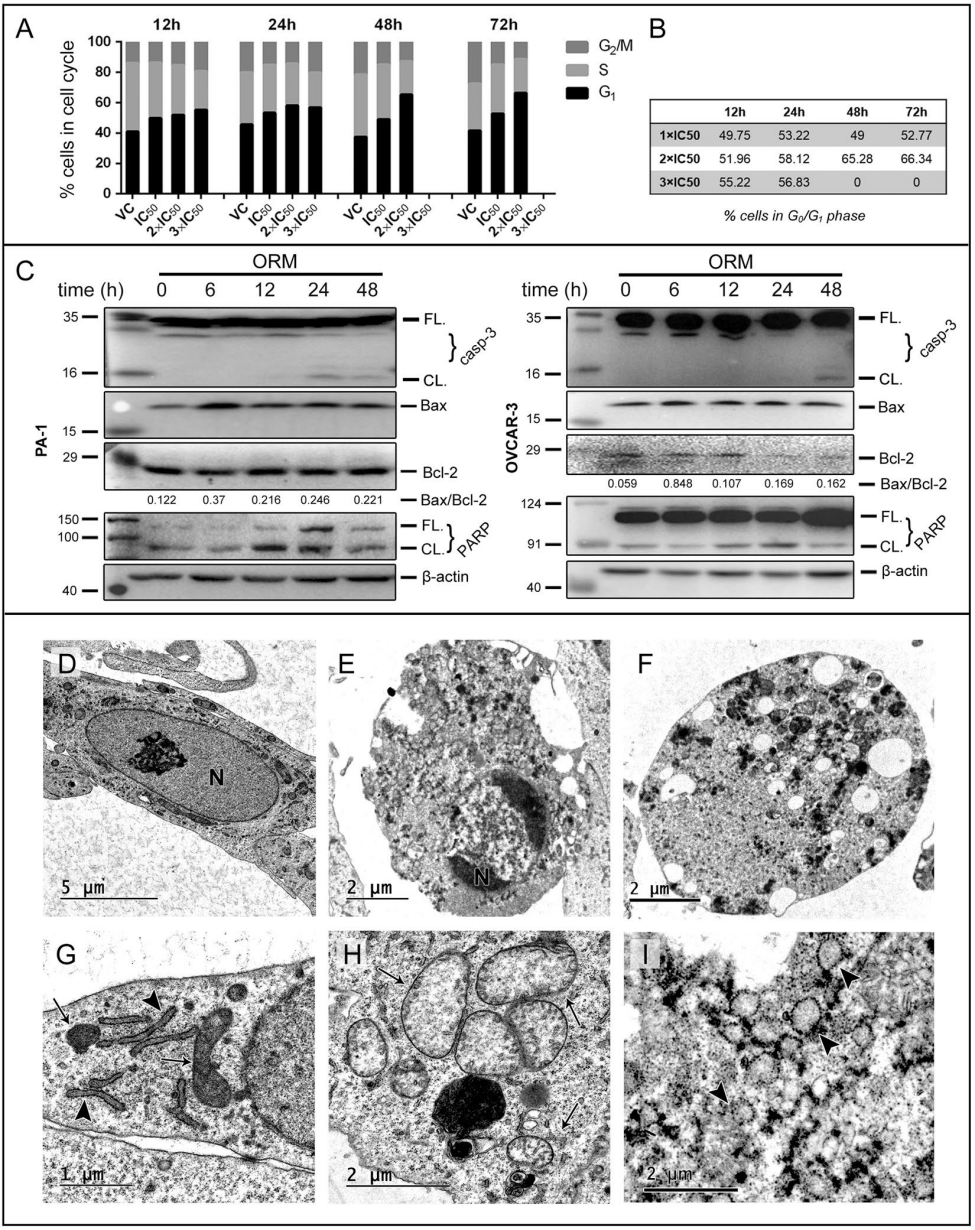
ORM induces caspase-dependent apoptosis. (**A**) Cell cycle analysis by flow cytometry of PA-1 cells post-treatment with ORM for indicated time (12 h, 24 h, 48 h & 72 h) and dose (1, 2 & 3 × IC_50_). (**B**) Percentage of cells in G_0_/G_1_ phase calculated for each time and dose. (**C)** PA-1 and OVCAR-3 cells were treated with ORM IC_50_ dose for indicated time periods and immunoblotted for apoptotic markers caspase 3, PARP, Bcl-2 and Bax. (**D**) Representative electron micrograph of control PA-1 cytoplasm, (**E**) early apoptotic cell (following treatment of IC_50_ dose of ORM) characterized by nuclear condensation, and (**F**) late apoptotic cell showing severe vacuolation. (**G**) Control mitochondria and ER of PA-1 cells; (**H**) swollen mitochondria and, (**I**) swollen ER post-treatment. N = nucleus, arrowheads = RER, arrows = mitochondria.

To distinguish between ORM-induced cell death as following a type-I (apoptotic) or type-II (autophagic) cell death pathway, we verified expression of apoptotic markers PARP, Bax and Bcl-2. Treatment with ORM arrested PA-1 cells at G_0_/G_1_ phase of the cell cycle (Fig. 3A,B), which corroborates with earlier studies in other cancer types^3,5^. Cleavage of PARP following treatment was evident in both cell lines, as was activation of caspase-3 (Fig. 3C). Further, increased pro-apoptotic Bax expression was observed while anti-apoptotic Bcl-2 levels remained unchanged or slightly declined, leading to increased Bax/Bcl-2 ratio (~2 fold increase in PA-1; ~3 fold increase in OVCAR-3 at 48 h) which is a marker for intrinsic apoptosis (Fig. 3C). Furthermore, examination of subcellular ultrastructure after 48 h of treatment in PA-1 revealed rounding and swelling of cells with increased vacuolation, pyknosis of the nucleus (Fig. 3E,F), mitochondrial swelling and disruption of cristae (indication of stressed mitochondria; Fig. 3H) and swelling of ER (indicating stressed ER; Fig. 3I) compared to control cytoplasm (Fig. 3D), and control mitochondria and ER (Fig. 3G) respectively. Moreover, ORM significantly depleted the mitochondrial membrane potential (Δψ_m_), which is responsible for release of cytochrome-c from mitochondrial intermembrane space into the cytosol. Cytochrome-c initiates the caspase activation cascade and is considered a ‘point-of-no-return’ for the intrinsic apoptotic pathway^20^. This was demonstrated with JC-1 staining in PA-1, where fluorescence shifted from aggregate form (590 nm) in healthy cells with intact Δψ_m_, to monomeric form (530 nm) in cells with depleted Δψ_m_ (Supplementary Fig. S1).

### Inhibition of ORM-induced autophagy by genetic or pharmacological methods promotes cell survival and suppresses apoptosis

**Figure 4:**
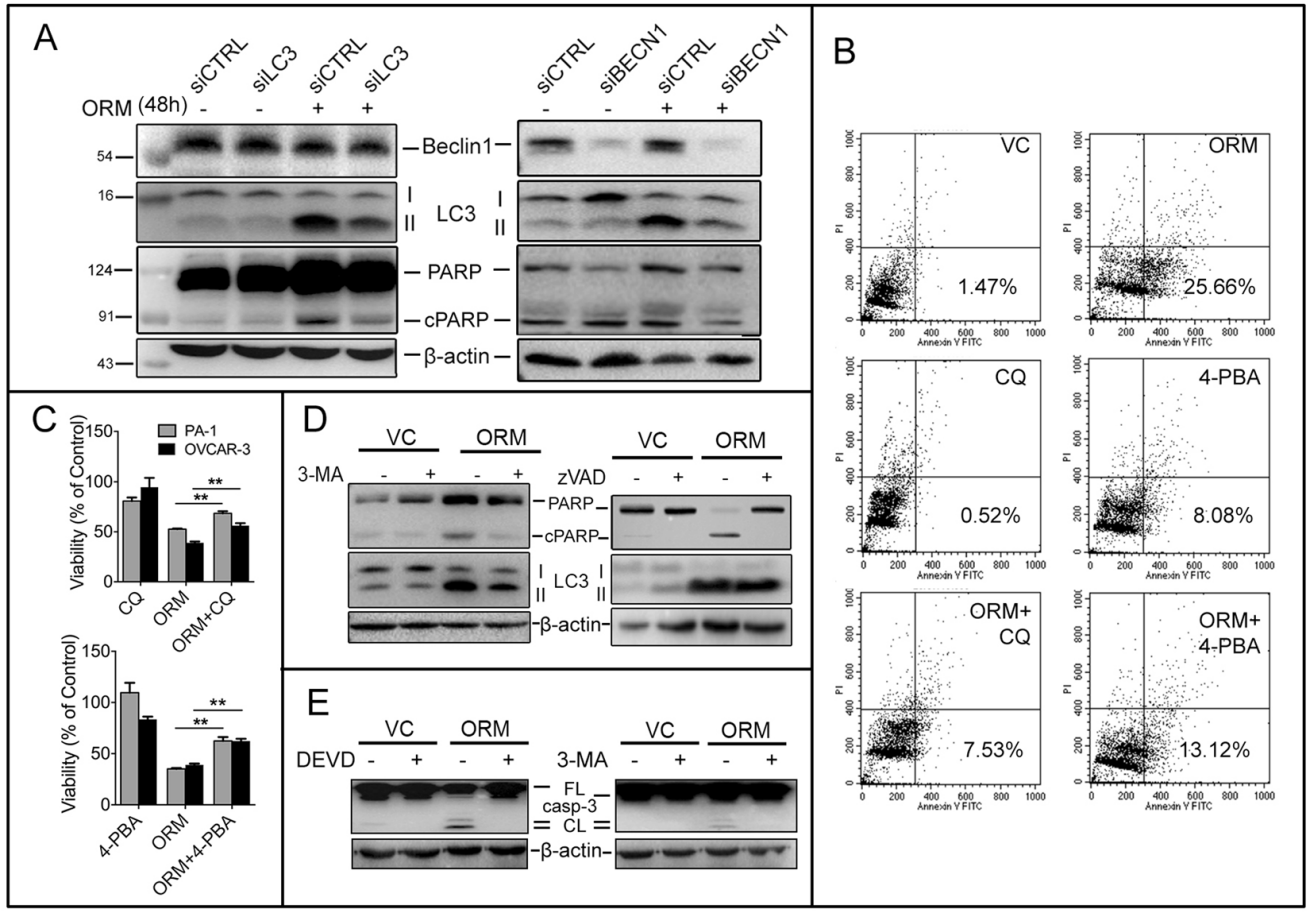
ORM-induced autophagy is upstream of apoptosis and pro-death in nature. (**A**) PA-1 cells were transfected for siRNA against either LC3 or Beclin 1, two critical autophagy proteins and cellular levels of LC3-II and cPARP were observed as markers of autophagic and apoptotic activity. (**B)** PA-1 cells were treated with ORM alone (IC_50_ dose), or pre-treated with lysosomal fusion inhibitor chloroquine (CQ; 10 μM, 2 h) or chemical chaperone and UPR inhibitor 4-PBA (5 mM; 2 h) before ORM treatment, stained with Annexin V-FITC/PI and analyzed by flow cytometry to assess apoptotic cell death (lower right quadrant = %early apoptotic population). (**C**) Viability (percentage of control) of PA-1 and OVCAR-3 cells pre-treated with CQ or 4-PBA and treated with ORM for 48 h measured through SRB assay; (**D**,**E**) PA-1 cells were pre-treated with 2.5 mM 3-MA or with 20 μM z-VAD-fmk or with 20 μM z-DEVD-fmk followed by 24 h ORM treatment (3-MA, z-DEVD) or 48 h ORM treatment (zVAD). PARP and LC3-II levels were analysed by immunoblotting.

Beclin 1 combines with mVps34 and mVps15 to form the pre-autophagosomal structures (PAS)^15^. This nucleation is followed by elongation, which is promoted by the Atg5-Atg12 cascade to conjugate LC3-I to PE. As previously discussed, ORM elevated Beclin 1 expression, along with an associated rise in Atg5-Atg12 which peaked at 24 h followed by a decline (data not shown), following the LC3-I → LC3-II conversion trend. Since Beclin 1 has crosstalk with the intrinsic apoptotic pathway (such as Bcl-2 binding to Beclin 1 through its BH3 domain), we checked whether silencing of cellular Beclin 1 affects apoptosis. siRNA against Beclin 1 reduced LC3-II levels as well as PARP cleavage (Fig. 4A, right). The result prompted us to check whether the reduction in PARP cleavage was solely due to Beclin 1 (and its crosstalks) or due to a general inhibition of autophagy. Similar decrease in PARP cleavage was observed when LC3 knockdown was performed, however it failed to decrease Beclin 1 level (Fig. 4A, left). This is expected, as Beclin 1 acts upstream of LC3 in autophagy and likely has more control on cell viability through its multiple interaction partners. We next sought to establish the interaction between autophagy and apoptosis induced by ORM. We found that- i) inhibiting autophagy by CQ pre-treatment (10 μM) induced a significant reduction in apoptotic cell population (PA-1; Fig. 4B) and increased cell viability (PA-1 and OVCAR-3; Fig. 4C, left); ii) inhibition of apoptosis by z-VAD-fmk increased cell viability (data not shown) and suppressed PARP cleavage, while it could not alter LC3-II conversion. On the other hand, suppression of autophagy by 3-MA was able to reduce PARP cleavage (Fig. 4D). It was also observed that activation of caspase 3 by ORM was inhibited by Z-DEVD-FMK and 3-MA (Fig. 4E). The data imply that autophagy is an upstream event of apoptosis. This observation is supported by reduced LC3-II levels at 48 h post-treatment compared to 24 h, signifying a decline in autophagy. The obervations collectively suggest that inhibition of autophagic flux enhances cell survival, and, autophagy thus induced by ORM is pro-death in nature.

### ORM-induced autophagy is generated by ER stress and unfolded protein response

**Figure 5:**
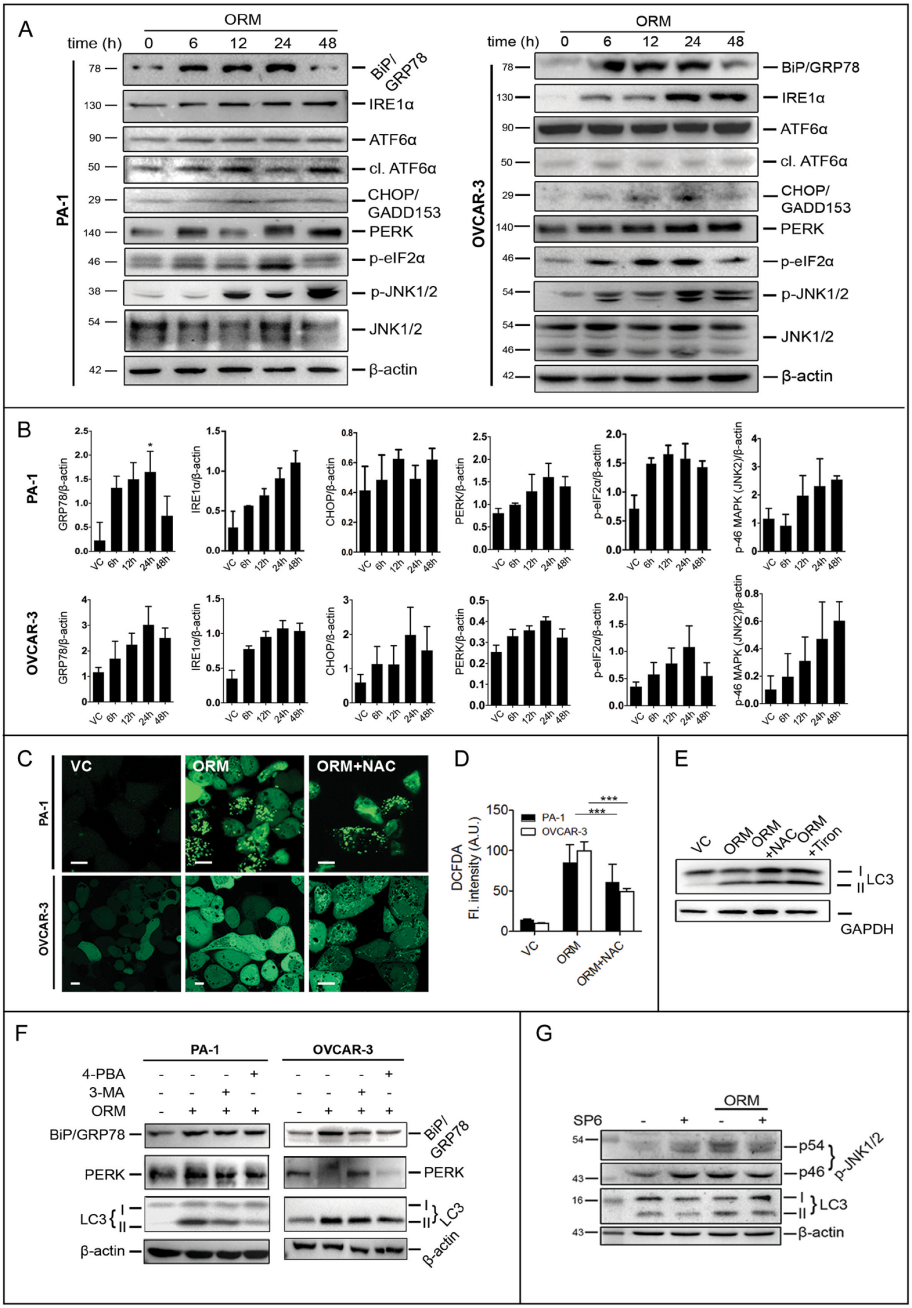
Upregulation of UPR proteins activate ER stress response, generation of reactive oxygen species and JNK activation, which in turn regulate autophagy. (**A**) PA-1 and OVCAR-3 cells were treated with ORM IC_50_ dose for indicated time-points and immunoblotted for UPR sensor proteins PERK, ATF6 and IRE1 as well as ER chaperone GRP78/BiP, downstream transcription factor CHOP/GADD153 and eukaryotic translation initiation factor eIF2α, apart from phosphorylated JNK (p-JNK) levels. (**B**) densitometric analyses of data represented in (**A**). (**C**) PA-1 and OVCAR-3 cells were grown in confocal glass-bottom dishes, treated with IC_50_ dose of ORM for 24 h followed by staining with CM-H_2_DCFDA, a live-cell ROS marker, and imaged in confocal microscope. (**D**) Quantification of fluorescence intensity of each cell from data such as (**B**) as described in Methods. (**E**) PA-1 cells were pre-treated with NAC (5 mM, 2 h) or Tiron (5 μM, 1 h), treated with ORM (24 h) and LC3 conversion was analysed by western blotting. (**F**) PA-1 and OVCAR-3 cells were pre-treated or not with 4-PBA (5 mM; 2 h) or 3-MA (2.5 mM; 2 h) before treating with IC_50_ dose of ORM and immunoblotting for GRP78 and PERK for quantification of ER stress and LC3 for quantification of autophagy. (**G**) PA-1 cells were pre-treated or not with small-molecule JNK inhibitor SP600126 (SP6; 10 μM,1 h) before IC_50_ ORM treatment and immunoblotted for LC3 as a marker of autophagy. (**E**) Scale bars = 10 μm.

Since ER stress is an established inducer of both autophagy and apoptosis^21,22^, we investigated ER stress by examining the Hsp-like ER chaperone BiP/GRP78 and the sensor proteins IRE1α, PERK and ATF6 present on the ER membrane. IRE1α is a major component and regulator of the UPR pathway^23^. Upon UPR activation, IRE1α splices X-box binding protein-1 (Xbp1) mRNA into its active form (a transcription factor); ATF6 is translocated to Golgi and cleaved by Golgi proteases while autophosphorylated PERK phosphorylates eIF2α, initiating what is known as an integrated stress response to counter oxidative stress and misfolded protein load^24^. These downstream factors are responsible for transcription of UPR target genes to combat misfolded protein load. C/EBP homologous protein (CHOP; also known as growth arrest and DNA-damage inducible gene-153, GADD153) is one such protein that can serve to counter UPR as well as initiate apoptosis, if necessary^22^. Figure 5A,B shows upregulation of the ER chaperone BiP/GRP78 along with the activation of transmembrane UPR sensors IRE1α, ATF6 (by cleavage) and PERK, besides elevated expression of CHOP/GADD153 in PA-1 and OVCAR-3. PERK phosphorylation (appearing as a migration of the band in Western blots^25^) is a major step in UPR and mediates the phosphorylation of eIF2α, which has a critical role in LC3 lipidation^26^. eIF2α was found to significantly increase up to 24 h, followed by decrease. A similar pattern of LC3-II turnover earlier (Fig. 1G,H) indicates eIF2α may indeed regulate the process and provides a possible link between autophagy and UPR.

Free radical generation under stress is a typical cellular response and is intrinsically tied to mitochondrial dysfunction. Furthermore, cellular ROS is an important initiatior of autophagy^27,28^. Indeed, treatment of N-acetylcysteine (NAC), a ROS quencher restored antiestrogen sensitivity in ROS-dependent autophagic resistance against antiestrogens^29^. We tested these possibilities by- i) measureing cellular ROS using CM-H_2_DCFDA staining and confocal microscopy, and, ii) checking LC3-II levels in presence of NAC. DCFDA staining revealed a significant increase in fluorescnce with 24 h treatment (mostly appeared to be localized in mitochondria), which was significantly reduced in presence of NAC (Fig. 5C,D). LC3-II levels did not significantly change when cells were co-incubated with the free radical quenchers NAC or tiron (Fig. 5E), suggesting no significant role of ROS generation in ORM-induced autophagy.

### ORM-induced autophagy is depdendent on UPR and works through activation of JNK

Since JNK inhibition suppressed autophagy, we wanted to evaluate the direct influence of UPR on autophagy. As seen in Fig. 5F, co-treatment of ORM and 4-PBA, a pharmacological UPR inhibitor^30^, in PA-1 and OVCAR-3 cells, reduced LC3-II turnover as well as a marked reduction of LC3 puncta in PA-1 (Supplementary Fig. S2), implying that inhibiting ER stress significantly impedes autophagy. 4-PBA pre-treatment increased viable cell population and rescued apoptotic cells (Fig. 4B,C), indicating that reduction of UPR-induced autophagy contributed to cell survival and reduced apoptosis. A reduction in cell viability was observed when PA-1 cells were treated with tunicamycin (a known UPR-inducer) alongwith ORM, suggesting ER stress indeed facilitated cell death (data not shown). 4-PBA also reduced overall UPR burden, as evidenced by overall reduction in BiP/GRP78 and PERK levels. Conversely, GRP78 and PERK levels were largely unaffected with 3-MA pre-treatment (Fig. 5F). Taken together, the results indicate UPR response upstream of autophagic induction by ORM, and that UPR induction by ORM contributes to the pro-death nature of autophagy.

To further explore the connection between the two processes (UPR and autophagy), we examined the activation of JNK1/2, which is a known IRE1α substrate and links UPR with apoptosis and autophagy^31^. p-JNK1/2 (at Thr183/Tyr185) was found to be highly upregulated on ORM exposure (Fig. 5A,B). This prompted us to examine how inhibition of JNK activity could influence autophagy and UPR. Pre-treatment of PA-1 cells with SP600125 (SP6), a small molecule JNK inhibitor, reduced GRP78 protein levels moderately (data not shown) while partially affecting LC3-II turnover (Fig. 5G). Indeed, GRP78 is known to be modulated by JNK activity and repressed when JNK was inhibited^32^. The data signifies autophagy suppression by inhibiting JNK, and establishes a correlation between JNK activity and autophagic turnover. The partial suppression of autophagy by SP6 may possibly be due to two other ER sensors (PERK and ATF6) that remain activated irrespective of IRE1-JNK.

### The Akt/mTOR axis is disrupted with ORM treatment

**Figure 6:**
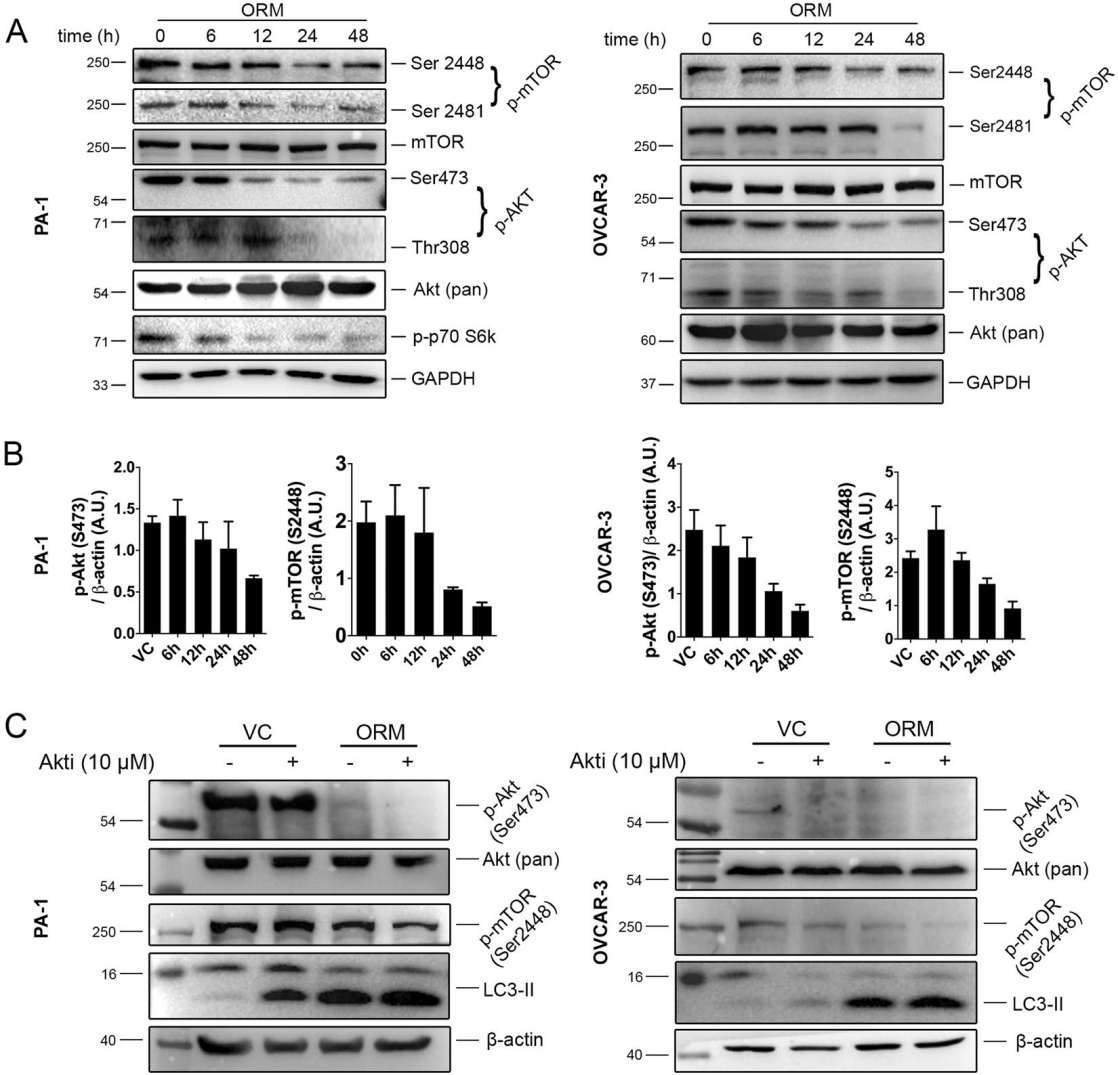
The Akt/mTOR pathway is involved in ORM-induced autophagy. (**A**) PA-1 and OVCAR-3 cells were treated with ORM IC_50_ dose for indicated time-points and immunoblotted for mTOR pathway proteins mTOR (phosphorylation sites at Ser2448 and Ser2481), Akt (phosphorylation sites at Ser473 and Thr308) and p70 S6k (at Thr389; in PA-1). (**B**) densitometric analyses for p-Akt (Ser473) and p-mTOR (Ser2448) from data represented in (**A**). (**C**) PA-1 and OVCAR-3 cells were treated with ORM alone or in combination with Akti (inhibitor of Akt 1/2; 10 μM) for 24 h and cellular levels of p-mTOR (as a downstream regulator of autophagy) and LC3 were observed by immunoblotting.

mTOR is one of the key players regulating autophagy during cellular stress and Akt/mTOR deactivation is necessary for autophagic process^33^. Akt is activated through phosphorylation at Thr308 and Ser473 in response to growth signal. Investigating the effect of ORM on Akt/mTOR pathway, we checked expression of phospo-Akt at these phosphorylation sites. Figure 6A shows steady decrease in Akt phosphorylation at Ser473 and Thr308 with time suggesting suppression of Akt activity in both PA-1 and OVCAR-3. Likewise, reduction in phosphorylated mTOR, downstream of Akt (at phosphorylation sites Ser2448 and Ser2481), was also seen upon treatment while level of total mTOR remained unchanged (Fig. 6A,B). A major downstream molecule of the mTOR axis is p70 S6 kinase, which, activated by mTOR, phosphorylates the S6 ribosomal protein to initiate protein synthesis. Phosphorylated (at Thr389) form of p70 S6k was also found to be steadily decreased (in PA-1). Regulation of autophagy by Akt kinase independent of mTOR (by reduced phosphorylation of Beclin 1) has previously been reported in starvation^34^. To check the role of Akt in ORM-induced autophagy, we inhibited Akt phosphorylation by pharmacological inhibition of Akt (Akt1/2 kinase inhibitor; Sigma). Combination of ORM and Akti resulted in additional LC3-II conversion in PA-1 and distinct downregulation of p-mTOR (Ser2448) in both PA-1 and OVCAR-3 (compared to ORM alone) (Fig. 6C). Since pharmacological Akt inhibition and ORM treatment follow an additive effect on mTOR regulation and autophagy, it highlights that direct disruption of Akt by ORM is a key effector in this type of autophagy.

### ORM inhibits ovarian tumor growth and induces autophagy *in vivo*

**Figure 7:**
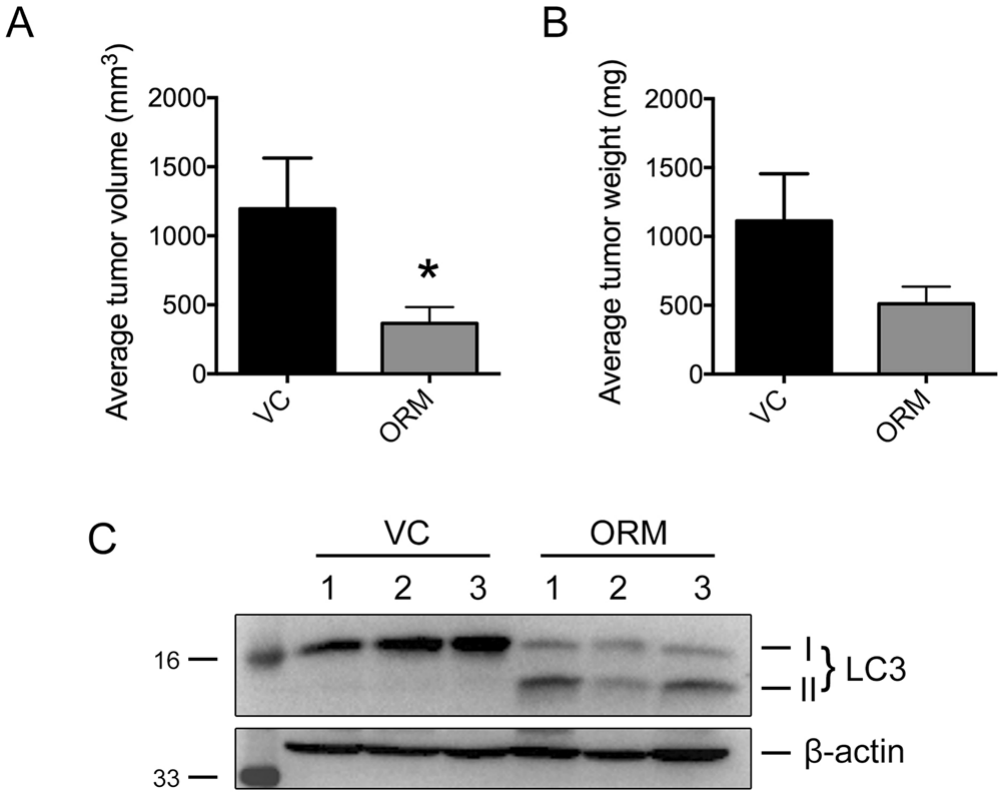
ORM administration reduces tumor progression and promotes autophagy *in vivo*. Reduction in both (**A**) tumor volume, and, (**B**) tumor mass was achieved when ORM (50 mg/kg body weight) was administered for 4 weeks. (**C**) LC3-II induction as a marker for autophagy activity in tumors isolated from control (VC) and ORM-treated (ORM) mice.

We carried out *in vivo* studies in ovarian xenograft model using PA-1 cells. As shown in Fig. 7A, intra-tumoral injection of ORM at 50 mg/kg dose caused a decrease in tumor size after 4 wks of treatment regimen and there was a significant (p < 0.05) difference in tumor volume between treated and untreated groups. Equally, there was marked reduction of mean tumor weight in ORM treated animals as compared to controls (Fig. 7B). Consistent with our *in vitro* results, immunoblotting of protein extracts from harvested tumor tissues revealed considerable induction of LC3-II upon ORM treatment in comparison to vehicle treated group (Fig. 7C), suggesting an obvious role of autophagy in anticancer activity of ORM.

## Discussion

Cancer cells, by virtue of their hypoxic, nutrient-poor environment are especially proficient in autophagic cell survival. However, the idea of an ‘autophagy-to-apoptotic switch’ has recently gained prevalence: a strong, irreversible autophagic response is shifted to programmed cell death by factors such as Beclin 1, Ambra 1, p38 MAPKs etc.^35,36^. In our study, we find that autophagy has a direct role in ORM-induced cell death; knockdown of autophagy genes Beclin 1 (functional autophagy protein) and LC3 (structural autophagy protein) by RNAi reduced PARP cleavage as well as pharmacological inhibition by CQ increased cell viability. Suppression of PARP cleavage by inhibiting autophagy can only indicate autophagy directly modulating apoptotic outcome by the crosstalk discussed above, because inhibiting autophagy by CQ increased cell viability and decreased apoptotic cell population. Tamoxifen, another SERM widely used against gynecological cancer, is a potent inducer of autophagy. Indeed, autophagy-dependent apoptosis has been noted for several tamoxifen analogs^37–39^. This is the first report of autophagy induction by ORM and indicates that most if not all SERMs have a propensity to activate autophagy to facilitate cell death. Interestingly, antiestrogen resistance in breast cancer is often attributed to pro-survival autophagic rescue of cells, along with inhibition of caspase-dependent apoptosis^40–42^. The present study appears to reveal a different mechanism of action of a SERM, with important differences such as – i) blockade of autophagy increases cell death by apoptosis, leading to restored antiestrogen sensitivity, while in case of ORM, blocking autophagy confers cell survival, ii) the mechanism of action of ORM may be independent of the action of estrogen receptor, as PA-1 is devoid of EsR^43^. ER-independent mechanism of autophagy by tamoxifen has been noted earlier for retinal pigment epithelial (RPE) cells^44^. Moreover, a tamoxifen analog, Ridaifen-B, has been shown to activate a non-canonical autophagy independent of the action of Beclin 1 in EsR-negative Jurkat cells^45^, indicating autophagy induction by SERMs may not be dependent on EsR. Furthermore, it was observed that ROS, a known autophagy initiator^46^ had no influence in ORM-induced autophagy.

Endoplasmic reticulum stress (ER stress) and unfolded protein response (UPR) is a major regulator of autophagy. UPR activation occurs through ER transmembrane proteins PERK (EIF2AK3), IRE1α and ATF6, which carry the response downstream to induce transcription of chaperone proteins to salvage misfolded protein load^47^. In normal circumstances, they are inactivated through their association with BiP/GRP78. Severe and prolonged UPR signal however, redirects to caspase-mediated apoptosis. Inactivation of the cellular mTOR/Akt axis is closely associated with autophagy^48^; mTORC1 is known to be inhibited by the MAP kinases ERK (extracellular signal regulated kinase) and JNK.

Induction of UPR upregulated IRE1α, which, in conjugation with JNK is necessary to induce autophagy by ER stress. As expected, the JNK inhibitor SP600125 moderately decreased GRP78 levels besides reducing LC3-II conversion, thus mitigating overall UPR (data not shown). IRE1α being a known inducer of JNK in periods of ER stress, it is possible that most of the UPR response might be dependent on IRE1α-JNK activity^22^. JNK activation is common during periods of ER stress and has recently been reported as an attractive target to enhance sensitivity of ovarian cancer to conventional chemotherapy^49^. Also, activation of JNK is frequently observed in relation to mTORC1 dephosphorylation and subsequently autophagy^31^. Additionally, inhibition of UPR by 4-PBA abrogated autophagic response (LC3-II conversion) and augmented cell survival/reduced apoptosis. This can only be due to 4-PBA directly thwarting the UPR control on autophagy to initiate apoptosis and not by non-specific reduction of misfolded protein load bypassing the autophagy pathway. A supporting evidence of the UPR-autophagy crosstalk comes from the activation of phosphorylated form of eIF2α as well as CHOP/GADD153 by ORM, which has been shown to act in concert with ATF4 to direct transcription of essential autophagy genes such as Becn1, Map1lc3b, Atg12 etc. in response to stress^43^. Interestingly, a study of autophagy-apoptosis crosstalk has been reported to regulate through GRP78, which confers resistance to antiestreogen therapy for estrogen receptor-expressing breast cancers through activation of pro-survival autophagy^46^. In view of ORM-induced ER stress resulting in a pro-death autophagic response, this suggests GRP78 (and in general UPR) may take a dynamic approach in regulating autophagy and apoptotic outcome.

mTOR is regarded as master regulator of autophagy^50^. Phosphorylation of mTOR at Ser2448 and Ser2481 residues are essential for its activation while catalytic activity of Akt is dependent on phosphorylation at Thr308 and Ser473. The Akt/mTOR axis is also equally important in regulating apoptosis signalling. Ormeloxifene, in its native form or in nanoparticle encapsulated form, has been demonstrated to suppress Akt/mTOR signalling in order to exert anti-cancer activity^4,51,52^. In line with these findings, we observed suppression of Akt/mTOR which was evident with their reduced phosphorylation level along with decrease in phosphorylation of p70 S6 kinase. Moreover, Akt inhibition by ORM is evident with Akt inhibitor co-treatment. The precise mechanisms involved in regulating the functional outcome of autophagy in cancer cells in response to chemotherapeutics is not well understood and is an area of intensive research. Our results establish that ORM induces autophagy through ER stress that regulates mitochondrial apoptosis. The present study is critical for understanding how ORM-induced autophagy can be modulated for a better therapeutic response. Our *in vivo* studies also validate the anti tumor potential of ORM along with induction of autophagy in agreement with our *in vitro* data. The ability of ORM to activate the pro-apoptotic arm of UPR for the induction of apoptosis while simultaneously suppressing pro-survival function of autophagy alongwith a proven safety profile of an existing drug, makes ORM an attractive option in the treatment of ovarian cancer.

## Methods

### Reagents and antibodies

ORM was procured from HLL Lifecare Limited. JC-1, antibiotic, AlexaFluor 488 and 568 conjugated antibodies, Lipofectamine 2000 were purchased from Invitrogen; z-VAD-fmk, protease inhibitor cocktail and GAPDH antibody were from Millipore. FITC-conjugated Annexin V was from BD Pharmingen. Beclin 1 and scrambled siRNA were from Dharmacon. Antibodies for cleaved PARP, LC3, Caspase 3, IRE1α, PERK, GRP78/BiP, p-p70 S6k, mTOR, p-mTOR, p-Akt as well as LC3 siRNA were purchased from Cell Signaling Technology. Beclin 1, Atg5 and β-actin antibodies were from Sigma. Antibodies for ATF6, CHOP/GADD153 and all secondary antibodies were from Santa Cruz. Spurr embedding kit was from Ted Pella. All other chemicals were from Sigma.

### Cell culture, plasmid and transfection

PA-1 and OVCAR-3 human ovarian carcinoma cell lines were obtained from the American Type Culture Collection and cultured in DMEM (PA-1) or RPMI 1640 (OVCAR-3) supplemented with 10% FBS (Gibco) with 1% penicillin-streptomycin in a humidified incubator at 37 °C/5% CO_2_. Cell line authentication for PA-1 was performed with STR profiling. All experiments were performed with low passage cells (P ≤ 15). The siRNA targeting human Beclin 1^53^, human LC3 (Cell Signaling, #6212) and non-targeted sequence (Cat. # D-001810-10-20) obtained from Dharmacon, Inc., (Lafayette, CO, USA); ptfLC3 was a gift from Tamotsu Yoshimori (Addgene plasmid # 21074)^17^. Cells were transfected with siRNA oligonucleotides/plasmids using Lipofectamine 2000 as per standard protocol. Cells were seeded in 60 mm culture dish/6 well plates for 24 hr in culture medium. After 40–50% confluence, cells were transfected with respective siRNA and plasmid, subsequently ORM treatment for 24 hr. After incubation, cells were harvested for further analysis. Knockdown of genes were analyzed by immunoblot. In confocal microscopy of tfLC3 transfected cells, green vesicles were considered to be autophagosomes, and red vesicles were considered to be autolysosomes.

### Viability and cell cycle analysis

Cell viability was determined with SRB assay as described earlier^54^. Cells were seeded in 96 well tissue culture plates (seeding density 10^5^ cells/ml), treated with increasing concentrations of drug and incubated for 48 h. Thereafter, cells were fixed with 10% TCA (1 h at 4 °C) followed by staining with 0.5% SRB (in 1% acetic acid) and air drying. SRB crystals were dissolved in 1 M Tris-Cl and absorbance values were recorded at 515 nm. IC_50_ values were calculated from standard curve by GraphPad Prism software. For cell cycle analysis, cells were harvested and fixed with ice-cold 100% ethanol, followed by incubation in RNAse A (10 µM, 30 min) and propidium iodide (5 μg/ml, 10 min) and analysis by flow cytometry.

### Immunofluorescence microscopy and MDC staining

Immunofluorescence staining for LC3 was performed as described previously^28^. Briefly, cells were seeded in glass coverslips, grown to required confluence and treated. For staining, the cells were fixed (4% PFA in PBS), permeabilized (0.5% Triton X-100) and blocked with 2% BSA in PBS. Cells were incubated with anti-LC3 antibody (1:100 in PBS-2%BSA; Cell Signaling) overnight at 4 °C,washed thrice with PBS, incubated with secondary antibody (1:250) for 1 h and stained with Hoechst 33342 before mounting in glass slides and imaging in a confocal microscope. For MDC staining, live cells adhered to confocal glass bottom dishes were stained with 10 mM MDC for 15 min at 37 °C. Imaging was done using a Carl Zeiss LSM510 META confocal microscope with proper excitation and emission filters and a 63 × 1.4 N.A. oil DIC objective. ImageJ was used for quantification of LC3 and MDC puncta selecting the ‘Analyze Particles’ function for ≥25 cells for each parameter.

### Transmission electron microscopy

Cells were fixed with 2.5% glutaraldehyde in 0.1 M cacodylate buffer; pH 7.4, post-fixed with 1% OsO_4_ and encapsulated in agarose. This was followed by dehydration with ascending grades of ethanol, infiltration, embedding and polymerization in Spurr resin. Ultrathin sections (50–70 nm) were obtained using a Leica Ultracut UCT ultramicrotome, picked up on copper grids and dual-stained with uranyl acetate/lead citrate. Grids were observed under a JEOL JEM 1400 TEM at 100 kV and imaged using a Gatan Orius 830 CCD camera with Gatan DigitalMicrograph software.

### Western blotting

Cells were harvested by scraping with ice-cold PBS and lysed with high salt lysis buffer (25 mM HEPES, 0.4 M NaCl, 1.5 mM MgCl_2_, 0.2 mM EDTA, 1% NP-40) with protease and phosphatase inhibitors. Lysate was denatured with Laemmli sample buffer and equal amounts were run on tris-acrylamide gels and transferred to PVDF membranes (Amersham), blocked with 5% non-fat milk or 5% BSA in TBST and probed with appropriate antibodies. Blots were developed with Clarity ECL substrate (Bio-Rad) and documented on a Bio-Rad ChemiDoc XRS+ system. Densitometric analyses were performed with BioRad ImageLab software.

### Annexin V-FITC/PI staining

Cells were cultured in 60 mm diameter tissue culture dishes up to 50% confluence. Treatment was given as described for indicated time. Cells were harvested by trypsinization, washed twice with cold PBS and resuspended in binding buffer (0.01 M HEPES, 0.14 M NaCl, 2.5 mM CaCl_2_ in aquous solution, sterile filtered). 10^4^ cells per sample resuspended in 100 μl binding buffer was collected in a flow cytometry tube and stained with FITC-conjugated Annexin V (according to manufacturer protocol) and PI (2 μg/ml) for 40 min at dark. This was follwed by addition of additional 400 μl of binding buffer and analysis in BD FACSCalibur flow cytometer with isotype controls and settings.

### JC-1 and DCFDA staining

For JC-1 staining, adhered cells were trypsinized and washed once with PBS before staining. Live cells were stained with JC-1 (2 µM; 20 min) in HBSS at dark, quantified in a BD FACSCalibur flow cytometer and analyzed with CellQuest Pro software (BD). DCFDA staining was performed as reported earlier^13^. Briefly, cells were seeded in glass coverslips, grown to required confluence and treated with drug (with or without inhibitor). Live cells were washed once with PBS, stained with 6 µM CM-H_2_DCFDA (30 min at RT) and imaged in a confocal microscope. Fluorescence intensity was quantified in LSM Image Examiner software by selecting the ROI around ≥25 cells for each parameter.

### *In vivo* study in ovarian xenograft tumor model

All animal studies were conducted in accordance with the principles and standard procedures approved by the Institutional Animal Ethics Committee (IAEC) CSIR-Central Drug Research Institute (Lucknow, India). Briefly, 10^7^ PA-1 cells in 100 µl PBS were inoculated subcutaneously at the right flank of 4–6 wk. old female nude Crl: CD1-Foxn1^nu^ mice. Once tumor volume reached 50 to 100 mm^3^, mice were randomized and divided into experimental groups (n = 5). Tumor-bearing mice were subjected to have intratumoral injection with either vehicle alone (10% ethanol v/v in sterile water) or ormeloxifene (50 mg/kg body weight) for 4 weeks. Tumor volumes of experimental animals were calculated according to the formula V = 0.5 × W^2^ × L where W is width and L is length of the tumor. At the end of the study animals were euthanized, tumors were dissected and weighed. A portion of the tumor tissues were lysed with T-PER reagent supplemented with protease inhibitors and protein concentrations were determined by a BCA protein kit. Protein lysates were analyzed by immunoblotting as described in Western blotting section.

### Statistical analysis

Experiments were performed at least three times independently. Error bars represent standard error (SEM) of mean of replicates. Statistical analyses were performed on GraphPad Prism v. 6 using unpaired Student’s t-test. Differences were considered significant as *p* < 0.05 (*), <0.01 (**) and <0.001 (***).

## Electronic supplementary material

Supplementary Information
