# Supplementary material for: Ormeloxifene-induced unfolded protein response contributes to autophagy-associated apoptosis via disruption of Akt/mTOR and activation of JNK
Source: Sci Rep. 2018 Feb 2;8:2303. doi: 10.1038/s41598-018-20541-8 (PMC5797234; doi:10.1038/s41598-018-20541-8)
Supplement: Supplementary file 1 — Supplementary Information [file 41598_2018_20541_MOESM1_ESM.pdf]

Ormeloxifene-induced unfolded protein response contributes to autophagy-associated apoptosis via disruption of Akt/mTOR and activation of JNK

Arindam Bhattacharjee[1,4], Mohammad Hasanain[2,4], Manoj Kathuria[1], Akhilesh Singh[2], Dipak Datta[2,3], Jayanta Sarkar[2,3]* and Kalyan Mitra[1,3]*

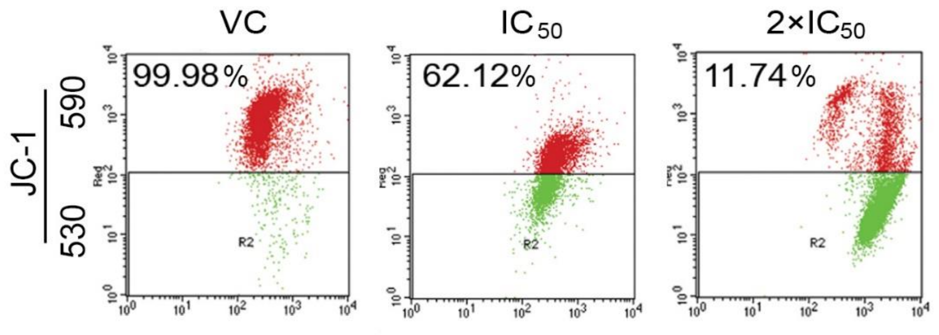

Supplementary Figure 1

PA-1 cells treated with IC50 or 2×IC50 dose of ORM for 48h time period were stained with potentiometric dye JC-1 (as described in Methods) and analysed in flow cytometry.

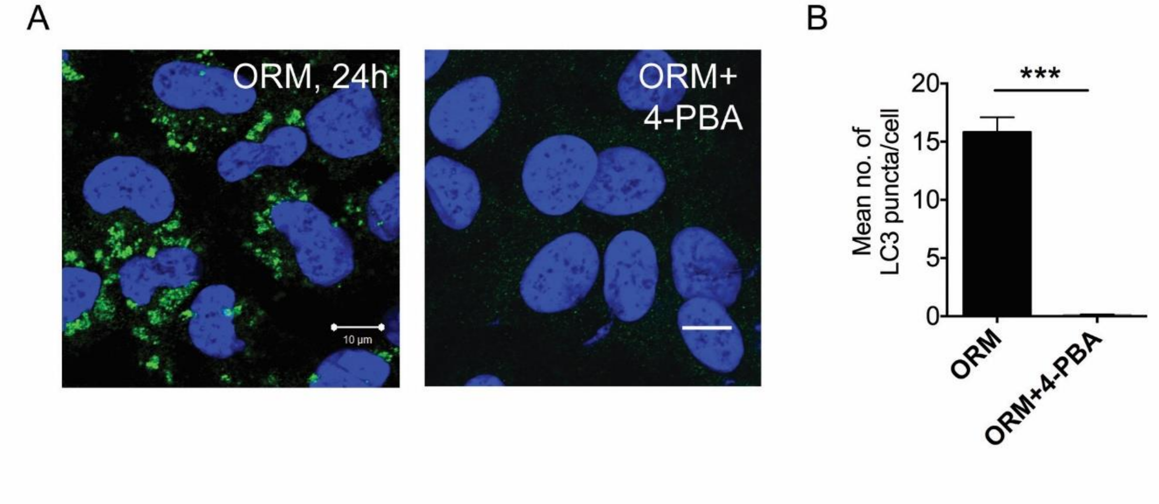

Supplementary Figure 2

(A) PA-1 cells pre-treated or not with 4-PBA (2.5 mM) were treated with ORM (24h), immunostained with LC3 and imaged in a confocal microscope. (B) quantification of LC3+ puncta/cell from data such as (B) as described in Methods.
